# Arginase 1: An Unexpected Mediator of Pulmonary Capillary Barrier Dysfunction in Models of Acute Lung Injury

**DOI:** 10.3389/fimmu.2013.00228

**Published:** 2013-08-07

**Authors:** Rudolf Lucas, Istvàn Czikora, Supriya Sridhar, Evgeny A. Zemskov, Aluya Oseghale, Sebastian Circo, Stephen D. Cederbaum, Trinad Chakraborty, David J. Fulton, Robert W. Caldwell, Maritza J. Romero

**Affiliations:** ^1^Vascular Biology Center, Medical College of Georgia, Georgia Regents University, Augusta, GA, USA; ^2^Department of Pharmacology and Toxicology, Medical College of Georgia, Georgia Regents University, Augusta, GA, USA; ^3^Division of Pulmonary Medicine, Medical College of Georgia, Georgia Regents University, Augusta, GA, USA; ^4^IDDRC/NPI, University of California Los Angeles, Los Angeles, CA, USA; ^5^Institute of Medical Microbiology, Justus-Liebig University, Giessen, Germany; ^6^Department of Anesthesiology and Peri-Operative Medicine, Medical College of Georgia, Georgia Regents University, Augusta, GA, USA

**Keywords:** pneumonia, capillary leak, pneumolysin, arginase 1, endothelial nitric oxide synthase

## Abstract

The integrity of epithelial and endothelial barriers in the lower airspaces of the lungs has to be tightly regulated, in order to prevent leakage and to assure efficient gas exchange between the alveoli and capillaries. Both G^−^ and G^+^ bacterial toxins, such as lipopolysaccharide and pneumolysin, respectively, can be released in high concentrations within the pulmonary compartments upon antibiotic treatment of patients suffering from acute respiratory distress syndrome (ARDS) or severe pneumonia. These toxins are able to impair endothelial barrier function, either directly, or indirectly, by induction of pro-inflammatory mediators and neutrophil sequestration. Toxin-induced endothelial hyperpermeability can involve myosin light chain phosphorylation and/or microtubule rearrangement. Endothelial nitric oxide synthase (eNOS) was proposed to be a guardian of basal barrier function, since eNOS knock-out mice display an impaired expression of inter-endothelial junction proteins and as such an increased vascular permeability, as compared to wild type mice. The enzyme arginase, the activity of which can be regulated by the redox status of the cell, exists in two isoforms – arginase 1 (cytosolic) and arginase 2 (mitochondrial) – both of which can be expressed in lung microvascular endothelial cells. Upon activation, arginase competes with eNOS for the substrate l-arginine, as such impairing eNOS-dependent NO generation and promoting reactive oxygen species generation by the enzyme. This mini-review will discuss recent findings regarding the interaction between bacterial toxins and arginase during acute lung injury and will as such address the role of arginase in bacterial toxin-induced pulmonary endothelial barrier dysfunction.

## Introduction

Cells require energy to carry out their vital functions. Mitochondrial oxidative phosphorylation is the main pathway through which cellular ATP is generated, provided that an adequate and continuous amount of O_2_ is supplied to the mitochondria. This is orchestrated by the microcirculation, which represents the interface between the parenchymal cells in the tissues (the consumers) and the circulatory system (the supplier).

In order for gas exchange between the 2^23^ alveoli and the lung capillaries in an adult human lung to occur in an optimal manner, the alveolar-capillary barrier integrity has to be tightly regulated (1). To that purpose, the continuous capillaries found in the lungs are very closely packed together. Since the rate of diffusion of O_2_ and CO_2_ through the alveolar-capillary barriers is proportional to the exchange area, but inversely proportional to its thickness, no excess leakage of liquid should occur in the interstitium and subsequently in the alveolar space, since this would dramatically impair gas exchange between the alveoli and the pulmonary capillaries. To that purpose, capillary hydrostatic pressure, which is partially defined by the pulmonary venous pressure and vascular tone, has to be kept under control, in order to prevent excess fluid extravasation from the capillaries.

## The Role of Arginase during Lung Inflammation: A Complicated Matter

Arginase, which converts l-arginine into l-ornithine and urea, is not only a key enzyme of the hepatic urea cycle, but is also expressed in extra-hepatic tissues lacking a complete urea cycle. Whereas cytosolic arginase 1 is the predominant isoform in the liver, mitochondrial arginase 2 is mainly expressed in extra-hepatic tissues (2). l-Arginine is not only a substrate for arginase, but can alternatively be converted to NO and l-citrulline by nitric oxide synthases (NOS). As such, arginase’s primary biological function in extra-hepatic organs, such as the lungs, lies mainly in the regulation of NO synthesis, by means of competing with NO synthase for the common substrate l-arginine.

**Figure 1:**
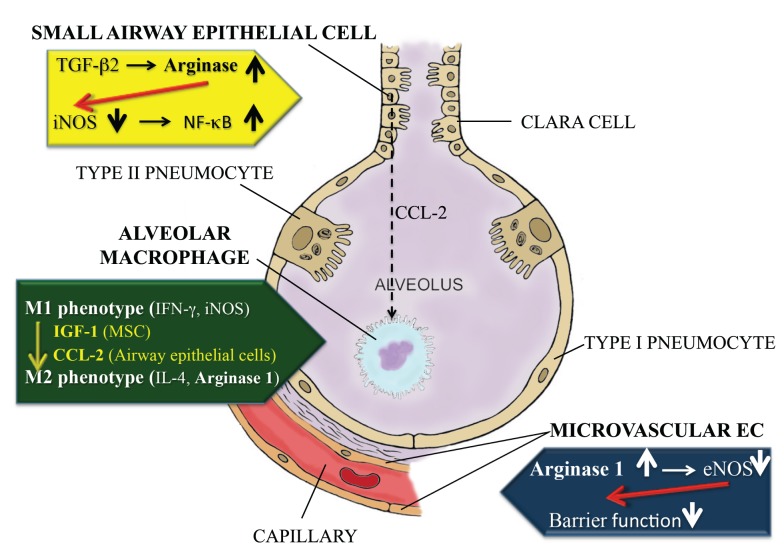
**Differential role of arginase in small airway epithelial cells, alveolar macrophages and capillary endothelial cells in the lungs**. Activation of arginase in the small airway epithelial cells (yellow panel) leads to a reduction in iNOS mRNA and protein expression, as such reducing NO generation. Since NO suppresses the NFκB response, this culminates in excessive NFκB activation and inflammation. Alveolar macrophages (green panel), upon stimulation with mesenchymal stem cell-derived IGF-1 or small airway epithelial cell-derived CCL2, can convert from a pro-inflammatory M1 phenotype, characterized by IFN-γ production and high iNOS activity, to an M2 phenotype, in which IL-4 generation and arginase 1 activity prevail. Activation of arginase 1 activity in microvascular endothelial cells (blue panel) leads to a reduction in eNOS-derived NO generation, which in turn contributes to a loss of barrier function in the capillary compartment.

Increased arginase activity has been reported in several inflammatory lung diseases, including asthma, chronic obstructive pulmonary disease, cystic fibrosis, and pulmonary hypertension. This suggests a common feature underlying the pathophysiology of these diseases [reviewed in (3)]. As demonstrated in Figure 1, both arginase 1 and 2 are constitutively expressed in airway and alveolar epithelial cells, endothelial cells, and alveolar macrophages in the lower airways (4, 5). However, the enzyme’s role can be quite different depending on the cell type or the location where its activity is increased.

## Preservation of Arginase Activity by Anti-Inflammatory Thioredoxin 1

The ubiquitously expressed and evolutionary well-conserved 12 kDa protein thioredoxin was initially thought to be primarily involved in protection against oxidative stress, by means of scavenging reactive oxygen species (ROS) through the interaction with peroxiredoxin and controlling the cellular redox balance (6, 7).

Thioredoxin exists as both a cytoplasmic and an extracellular form. In contrast to thioredoxin 2, thioredoxin 1 has the capacity to function as a chaperone for arginase, as such protecting the enzyme from inhibition by reactive oxygen and nitrogen intermediates and from denaturation by urea and heat. As such, thioredoxin 1 retains arginase in a catalytically active state (8).

Thioredoxin not only reduces oxidative and nitrosative stress, but also suppresses pro-inflammatory cytokine generation (9), reduces leukocyte-endothelial interactions and preserves arginase activity, which in turn blunts deleterious inducible NOS (iNOS) activity. Taken together, all of these activities make thioredoxin a potent and versatile mediator of inflammation (10, 11). Recombinant human thioredoxin was proposed as a therapeutic candidate for the treatment of several inflammatory disorders (12).

In the lungs, the induction of thioredoxin is regarded as an adaptive response against lung inflammation associated with oxidative stress (13). Recent studies have revealed that exogenously administered thioredoxin protects the lungs from acute lung injury induced by influenza virus infection (14). In conclusion, thioredoxin 1 has the capacity to preserve arginase activity in pulmonary cells, as such blunting excessive iNOS activity.

## Role of Arginase in Alveolar Macrophages during Pulmonary Infection and Inflammation

In alternatively activated alveolar macrophages of the M2 phenotype, arginase activity can limit the consummation of l-arginine by iNOS, as such suppressing the cytotoxic response by these cells (Figure 1). Many factors can affect the conversion from the M1 to the M2 phenotype in these cells.

Following *Mycobacterium tuberculosis* infection, alveolar macrophages first become classically (M1) polarized, characterized by an increased expression of IFN-gamma and iNOS (Figure 1). However, as the inflammation progresses, they decrease iNOS and IFN-γ expression, but increase IL-4 generation and arginase 1 activity, indicating M2 polarization (15). By contrast, *M. tuberculosis*-induced granuloma-associated macrophages remain M1-polarized throughout the entire process. Azithromycin treatment was shown to have the potential to induce M2 polarization of alveolar macrophages, e.g., in a *Pseudomonas aeruginosa* infection model (16).

The NO-generating capacity and arginase activity of alveolar macrophages also seems to affect susceptibility to infection with *Chlamydia*. Indeed, C57BL/6 mice develop severe pneumonia and poor immunity against *Chlamydia* after moderate respiratory infection, whereas BALB/c mice are protected from the disease and develop a vigorous Th1 response. Infected C57BL/6 macrophages release more iNOS-derived NO than BALB/c macrophages and express lower mRNA concentrations of arginase 2. Reduction of NO production upon incomplete iNOS inhibition abolishes susceptibility of C57BL/6 mice to *Chlamydia*-induced disease. Thus, the quantity of NO released by infected macrophages seems to define pathogenic versus protective macrophage responses to chlamydial infection (17).

In rat alveolar macrophages, bacterial lipopolysaccharide (LPS), released upon antibiotic treatment, was shown to increase both arginase 1 and 2 expression, an effect which could be blunted by glucocorticoids (5). It is important to note that arginase in alveolar macrophages not only contributes to impairment of NO generation, but it can also mediate airway remodeling, as detected in asthma, cystic fibrosis, and COPD, through the increased production of l-proline, a precursor of collagen, and the polyamines putrescine, spermidine, and spermine from l-ornithine (18).

The role of arginase in alveolar macrophages in LPS-induced acute lung injury remains controversial, since a recent study reported that mesenchymal stem cell-conditioned medium mediates the resolution of LPS-induced acute lung injury, by attenuating lung inflammation and promoting a wound healing/anti-inflammatory M2 macrophage phenotype, characterized by increased arginase 1 activity, at least partially in an insulin growth factor 1 (IGF-1)-dependent manner (19).

## Role of Arginase in Small Airway and Alveolar Epithelial Cells

From the previous paragraph, it is noteworthy that the switch of alveolar macrophages from the M1 to the M2 phenotype can be mediated by other cell types in the lower airways. This is not only the case for mesenchymal stem cells, but also for airway epithelial cells. Indeed, supernatants from chitin-treated airway epithelial cells were shown to induce alternative M2 activation of alveolar macrophages *in vivo*, by means of a CCL2 chemokine-dependent mechanism [Figure 1; (20)].

Moreover, TGF-β2 was shown to impact cytokine-induced NO production in primary small airway epithelial cells, by enhancing total arginase activity and reducing iNOS mRNA and protein levels, through a Rho kinase-dependent pathway (21). In a different study, reduction of arginase activity was shown to enhance the cellular content of NO and S-nitrosated proteins in a mouse type II alveolar epithelial cell line (22). As a consequence, TNF- or LPS-stimulated NF-κB DNA binding and transcriptional activity was decreased, in combination with an enhanced S-nitrosation of p50. The NOS inhibitor N-omega-nitro-l-arginine methyl ester (L-NAME) reversed the effects of arginase inhibition on NF-κB, suggesting a causal role for NO in the attenuation of NF-κB induced by arginase suppression. Conversely, overexpression of arginase 1 decreased cellular S-nitrosothiol content, enhanced IκB kinase activity and NF-κB DNA binding and decreased S-nitrosation of p50.

These results point to a regulatory mechanism wherein NF-κB is controlled through arginase-dependent regulation of NO levels, which may impact on chronic inflammatory diseases that are accompanied by NF-κB activation and upregulation of arginases (22, 23).

## Regulation of Capillary Endothelial Permeability

Endothelial cells form confluent monolayers on the surface of the inner wall of blood vessels. One of their major functions is therefore the separation of blood from underlying tissues, allowing only tightly controlled passage of macromolecules and cells. In the lower airways, where the actual gas exchange between the alveoli and the capillaries only efficiently occurs when barriers are tight (1), confluence is crucial. The adherence of endothelial cells is formed by transmembrane adhesion proteins, which mediate homophilic adhesion and junctional structures.

The transmembrane proteins are linked to specific intracellular partners, which mediate anchorage to the actin cytoskeleton and, as a consequence, stabilize junctions. The changes in the components of the EC cytoskeleton are of critical importance in the determination of the actual shape of the cell. The actin filaments and the phosphorylation/dephosphorylation-controlled actomyosin interactions are dramatically involved in the increase of the vascular permeability. Phosphorylation/dephosphorylation events of cytoskeletal/cytoskeleton-associated proteins also have a regulatory role in endothelial barrier regulation. Ca^2+^, calmodulin, and myosin light chain kinase (MLCK) were shown to be required components for endothelial cell retraction, whereas myosin phosphatase restores endothelial relaxation (24).

Two main signaling pathways regulate the barrier function, via the inhibition of myosin phosphatase. One of them is the vasoactive agent-induced Rho pathway, which increases the endothelial permeability. Rho Kinase (ROCK) may increase myosin light chain (MLC) phosphorylation indirectly by means of inducing myosin phosphatase inactivation, accumulation of diphospho-MLC, and cell contraction. Another pathway involves the Protein Kinase C (PKC)-potentiated inhibitory protein of 17 kDa (CPI-17), which may also affect isolated protein phosphatase 1c (PP1c) or the holoenzyme form of myosin phosphatase, without dissociating its subunits (25, 26).

External factors, such as certain G^+^ bacterial toxins (e.g., pneumolysin and listeriolysin) or pro-inflammatory cytokines, such as TNF, can induce increases in Ca^2+^-influx in endothelial cells, which can affect the microtubular network dynamics. Increases in intracellular Ca^2+^ can induce disassembly of microtubules (27). The microtubule population in endothelial cells is heterogeneous and can be divided into (1) stable, modified (acetylated), and (2) dynamic microtubules, with the former ones being more stable and thus more resistant to the effects of external factors. It is possible that under conditions compromising vascular endothelial integrity, the stable microtubules may confer stability to the endothelial microtubule network (28). Depolymerization of microtubules can in turn cause disassembly of adherens junction proteins with which they associate, such as VE-cadherin, thus increasing permeability (29).

## Restoring Capillary Barrier Function in Pneumonia: A Therapeutic Priority

Childhood pneumonia is the leading single cause of mortality worldwide in children aged less than 5 years (30). Moreover, over four million people develop pneumonia each year in the United States, with over a half a million of them being admitted to a hospital for treatment. Community-acquired pneumonia (CAP) represents a major cause of morbidity and mortality in mainly elderly patients (31, 32). The leading bacterial cause is the Gram-positive bacterium *Streptococcus pneumonia* (pneumococcus), being identified in 30–50% of pneumonia cases. The fatality rate associated with *Streptococcus pneumoniae*, the main etiological agent of severe pneumonia, still approximates 20%, despite the use of potent antibiotics and aggressive intensive-care support (30, 32).

Permeability edema, associated with severe pneumonia, acute lung injury, and the acute respiratory distress syndrome (ARDS), is characterized by a dysfunction of the alveolar-capillary barriers, leading to an infiltration of, e.g., neutrophils and factors contained in the blood into the alveoli. In severe pneumonia, this condition, characterized by capillary endothelial hyperpermeability, can occur days after initiation of antibiotic therapy, thus when tissues are already sterile. Permeability edema in pneumococcal pneumonia correlates with the presence of the bacterial virulence factor pneumolysin (33, 34), a cytoplasmic hemolytic protein released during bacterial autolysis or upon treatment with β-lactam antibiotics (35). Pneumolysin-induced acute lung injury was suggested to result from direct pneumotoxic effects on the alveolar-capillary barrier, rather than from resident or recruited phagocytic cells (36).

Intravascular pneumolysin was shown to cause a significant dose-dependent increase in pulmonary vascular resistance and in lung microvascular permeability. Upon binding of pneumolysin to cholesterol in cell membranes, oligomerization, and pore formation occur, which causes increased intracellular Ca^2+^ levels (37), the initial pivotal signal preceding pathways leading to endothelial cell contraction (38), including MLC-dependent mechanisms and microtubule rearrangement. RhoA and Rho-associated kinase may directly catalyze MLC phosphorylation or act indirectly via inactivation of myosin phosphatase to induce cell contraction and endothelial barrier disruption. In turn, endothelial barrier enhancement is associated with Rac 1-mediated formation of F-actin, increased association of focal adhesion proteins, and enlargement of intercellular adherens junctions. Thus, a precise balance between RhoA- and Rac1-mediated signaling is essential for endothelial barrier regulation.

The Ca^2+^-dependent PKC isoform, PKC-α, was suggested to play a critical role in initiating endothelial cell contraction and disassembly of VE-cadherin junctions (39–41). The NADPH oxidases, Nox2 and Nox4, are major sources of ROS in endothelial cells and are implicated in redox-sensitive signaling pathways that influence endothelial cytoskeletal organization and permeability (42, 43). Apart from inducing RhoA activation (41), PKC-α activation was also recently shown to upregulate Nox 4 mRNA expression in human endothelial cells (44).

In view of its crucial role in bacterial virulence and its profound effects on the immune system of the host, pneumolysin can be considered as a model toxin for G^+^ infection-associated acute lung injury and permeability edema. Since no standard therapy is currently available to treat the pulmonary permeability edema associated with severe pneumonia, the need for novel substances that can improve oxygenation in these patients, by means of barrier restoration is thus very important.

## An Important Role for Arginase 1 in Pneumolysin-Mediated Capillary Leak

In the larger blood vessels, impaired vasorelaxation capacity can be caused by dysfunctional endothelial nitric oxide synthase (eNOS)-dependent NO generation and increased eNOS-dependent ROS production. Moreover, in acute lung injury, the nitrating agent peroxynitrite, which can be formed by the reaction between ROS and NO in severe pathophysiological situations, can further deteriorate the vascular dysfunction (45). Endothelial NOS dysfunction in large vessel endothelial cells can be induced upon arginase 1 activation (46, 47). Although it is generally recognized that NO is a crucial regulator of vessel vasodilation, its role in the capillaries is less well documented, apart from its anti-adhesive and anti-aggregatory effects on platelets (48).

Maintenance of the endothelial barrier requires a basal level of NO, regulated by eNOS (49). Both the lack of NO and high NO levels destabilize inter-endothelial junctions (50–52). Availability of the semi-essential amino acid l-arginine is required for eNOS activity and NO production and is essential for vascular integrity and function. Recent studies have indicated that increased activity and/or overexpression of the enzyme arginase, which is thought to be dependent on RhoA activation, may play an important role in the availability of l-arginine and thus in the pathogenesis of vascular dysfunction (46, 53). Both arginase 1 and 2 have been found in endothelial cells, with arginase 1 being the dominant isoform. Arginase competes with eNOS for their common substrate, l-arginine, thus reducing the NO-generating capacity of the enzyme. Endothelial NOS catalyzes the two step conversion of substrate l-arginine into NO and utilizes electrons from NADPH to reduce molecular oxygen followed by oxidation of the guanidino N group of arginine to form NO, l-citrulline, and water (54).

It is this repeated activation of molecular oxygen that presents the opportunity for enzymatic missteps and superoxide production from eNOS, in the presence of reduced l-arginine availability, a process that has been termed “eNOS uncoupling” (54, 55). All of these events will culminate in endothelial hyperpermeability.

Our recent studies have shown that treatment of human lung microvascular endothelial cells with a sublytic concentration of pneumolysin, within 2 h significantly increases both arginase 1 and 2 expression, as well as arginase activity (56). This is accompanied by a significant reduction in basal NO generation and hyperpermeability in these cells. Both the barrier function and the NO generation can be partially restored upon treating the cells with the arginase inhibitor (S)-(2-boronoethyl)-l-cysteine (BEC) or with the PKC-α inhibitor Ro32-4032 *in vitro*. PKC-α signals RhoA activation (57), which in turn is involved in the activation of arginase activity in endothelial cells (58, 59).

Moreover, both the arginase inhibitor BEC and the PKC-α inhibitor Ro32-4032 can blunt pneumolysin-induced endothelial hyperpermeability in HL-MVEC *in vitro*. These results are substantiated *in vivo*, since arginase 1^+/−^/arginase 2^−/−^, but not arginase 1^+/+^/arginase 2^−/−^ mice (60) are significantly protected from pneumolysin-induced capillary leak (56).

Taken together, these results demonstrate an important role for arginase 1 in pneumolysin-induced barrier dysfunction in HL-MVEC.

## Manipulating Arginase Activity in Acute Lung Injury: Handle with Care

The previous paragraph points toward a potential therapeutic potential for arginase inhibitors in the treatment of capillary leak associated with acute lung injury, ARDS, and severe pneumonia. However, as previously discussed, the important role of arginase in the M2 phenotype of alveolar macrophages, which can, e.g., promote clearance of LPS-induced edema inflammation, makes a general inhibition of arginase activity in the lungs during infection problematic. Moreover, direct arginase inhibitors block arginase activity not only in the endothelium but also in other tissues, such as the liver, where its activity is needed in the urea cycle. As such, whereas arginase inhibition in capillary endothelial cells might increase their barrier function, it can potentially also lead to the conversion of alveolar macrophages from the protective M2 to the deleterious inflammatory M1 phenotype, the latter of which is characterized by excessive iNOS-mediated NO generation. Moreover, arginase inhibition in type II alveolar epithelial cells can also cause activation of iNOS and thus excessive NO production, shown to blunt the open probability of the epithelial sodium channel, crucial for alveolar liquid clearance during acute lung injury (61, 62). Furthermore, because arginase is involved in macrophage-mediated anti-bacterial responses and in wound repair (63), it is possible that substances inhibiting its activation, although conferring resistance to pneumolysin-mediated vascular leak, can interfere with macrophage-mediated innate immunity to pneumococci and wound healing. The same holds true for PKC-α proposed to be important for T-cell proliferation and IFN-γ production (64), and as such can be involved in anti-bacterial defense mechanisms, making that direct PKC-α inhibitors potentially can have negative effects on adaptive immunity.

Therefore, alternative substances interfering with the activation of arginase 1, rather than with its activity *per se*, preferentially in the endothelium, should be identified and investigated more in detail.

Although it is generally assumed that cytokines solely exert their activities upon activating their respective receptors, this does not seem to be true in the case of TNF. Apart from its receptor binding sites, which mediate a plethora of biological activities, ranging from apoptosis to inflammation and proliferation (65, 66), TNF exerts a lectin-like activity, permitting its binding to glycoproteins such as uromodulin, with a *K*_D_ = 10^−10^ M (67) uromodulin is a glycoform of Tamm–Horsfall protein, found in the loops of Henle of pregnant women, which was shown to bind the pro-inflammatory cytokines, IL-1β, IL-2, and TNF, proposed as a mechanism to clear excessive levels of these cytokines from the circulation during pregnancy (67, 68).

Since uromodulin-bound TNF was still able to exert cytotoxic effects in L929 fibrosarcoma cells, it was proposed that the lectin-like domain of TNF has to be spatially distinct from its receptor binding sites. Specific oligosaccharides, such as *N,N*′-diacetylchitobiose, as well as branched trimannoses, which are known to bind to the lectin-like domain of TNF, are able to inhibit the necrotic activity of TNF in African trypanosomes, but not the cytotoxic activity of TNF in L929 cells. Moreover, lectins with a similar oligosaccharide specificity as TNF, such as Urtica Dioica Agglutinin, but not those with a different specificity, block the trypanolytic effect of TNF (69). Molecular graphics comparisons of tertiary structures of TNF (trypanolytic) and the highly homologous lymphotoxin-α (non-trypanolytic), lead us to propose a dissimilar structure that could be responsible for the lectin-like activity. This structure, which is present at the Tip of the TNF molecule can be mimicked by a circular 17 amino acid peptide, which we called the TIP peptide. Antibodies to this peptide were able to inhibit the trypanolytic activity and moreover, the TIP peptide itself was shown to exert trypanolytic activity (69). Three amino acids, i.e., one threonine and two glutamic acids were shown to be crucial for this activity.

Our recent data have demonstrated that the TIP peptide inhibits pneumolysin-induced PKC-α and arginase activation and restores basal NO generation in human microvascular endothelial cells monolayers. The peptide moreover significantly protects from pneumolysin-induced capillary leak *in vivo* (56). Since the TIP peptide also activates lung liquid clearance upon stimulating epithelial sodium channel function, the latter of which is impaired by pneumolysin (70), it is further being investigated as an alternative to inhibitors of arginase and PKC-α in the treatment of permeability edema. As such, the peptide is being tested in two phase 2a clinical trials in patients with acute lung injury.

## General Conclusion

Recent studies have revealed a complex role for arginase during acute lung injury. On the one hand, activation of arginase 1 in alveolar macrophages, as can be induced by airway epithelial cells or mesenchymal stem cells, promotes the protective M2 phenotype in these cells, reducing deleterious iNOS activity. By sharp contrast, an increase in arginase 1 activity in pulmonary microvascular endothelial cells, as can be induced by bacterial toxins, can mediate capillary endothelial hyperpermeability, by means of reducing basal eNOS-dependent NO generation, crucial for the generation of basal barrier function. These findings thus clearly document the difficulty to develop therapies interfering directly with arginase activity during acute lung injury. As such, targeting arginase in specific cell types in the lungs, using, e.g., genetically modified mice with inducible constructs could lead to a better understanding of this complex area.

## Conflict of Interest Statement

The authors declare that the research was conducted in the absence of any commercial or financial relationships that could be construed as a potential conflict of interest.
